# The Effects of rs405509 on *APOEε4* Non-carriers in Non-demented Aging

**DOI:** 10.3389/fnins.2021.677823

**Published:** 2021-06-10

**Authors:** Dongpeng Wu, Han Zhao, Huali Gu, Bin Han, Qingqing Wang, Xu Man, Renliang Zhao, Xuejun Liu, Jinping Sun

**Affiliations:** ^1^Department of Neurology, The Affiliated Hospital of Qingdao University, Qingdao, China; ^2^Department of Radiology, The Affiliated Hospital of Qingdao University, Qingdao, China; ^3^Department of Emergency Internal Medicine, The Affiliated Hospital of Qingdao University, Qingdao, China; ^4^Institute of Integrative Medicine, The Affiliated Hospital of Qingdao University, Qingdao, China

**Keywords:** *APOEε4*, Alzheheimer’s disease, rs405509, amplitude of low-frequency fluctuations, degree centrality (DC), regional homogeneity (ReHo), fALFF (fractional amplitude of low frequency fluctuations), PerAF

## Abstract

**Background:**

There is evidence that the T allele of rs405509 located in the apolipoprotein E (*APOE*) promotor region is a risk factor for Alzheimer’s disease (AD). However, the effect of the T/T allele on brain function in non-demented aging is still unclear.

**Methods:**

We analyzed the effects of the rs405509 T/T allele on cognitive performances using multiple neuropsychological tests and local brain function using resting-state functional magnetic resonance imaging (rs-fMRI).

**Results:**

Significant differences were found between T/T carriers and G allele carriers on general cognitive status, memory, and attention (*p* < 0.05). Rs-fMRI analyses demonstrated decreased amplitude of low frequency fluctuation (ALFF) in the right middle frontal gyrus, decreased percent amplitude of fluctuation (PerAF) in the right middle frontal gyrus, increased regional homogeneity (ReHo) in the right cerebellar tonsil and decreased ReHo in the right putamen, and decreased degree centrality (DC) in the left middle frontal gyrus (*p* < 0.05, corrected). Furthermore, significant correlations were found between cognitive performance and these neuroimaging changes (*p* < 0.05).

**Conclusion:**

These findings suggest that T/T allele may serve as an independent risk factor that can influence brain function in different regions in non-demented aging.

## Introduction

Alzheimer’s disease (AD) is a degenerative disease of the brain characterized by progressive cognitive and behavioral impairment in the elderly and the early stages of old age (Ferri et al., 2005). Apolipoprotein E (*APOE*) is the gene most closely related to the occurrence of AD (Farrer et al., 1997). Compared with non-carriers, carriers of the ε4 allele of the *APOE* gene have been shown to be associated with a higher risk of developing Alzheimer’s disease in late-onset families (van der Flier et al., 2011). With the advance of neuroimaging techniques, there is increasing evidence that the *APOEε4* allele is related to changes in brain structure and function (Reiman et al., 2004; Heise et al., 2011; Machulda et al., 2011).

However, other *APOE* polymorphisms have been proved to affect the occurrence of AD, with the exception of the ε4 allele. The *APOE* promotor rs405509, also termed Th1/E47cs or −219 T/G, has a substantial impact on the expression of the *APOE* gene as well as the development of AD (Lambert et al., 1998b). The rs405509 According to previous reports, the *APOE* promotor’s T/T allele is a verisimilar risk factor for developing AD (Beyer et al., 2002). This polymorphism is also associated with myocardial infarction and has become a common risk factor for cardiovascular disease and neurodegenerative disease, particularly in aging (Lambert et al., 2000; Ye et al., 2003).

The rs405509 polymorphism is considered functional. As a regulator of *APOE* gene expression, it is likely to regulate specific transcriptional processes (Laws et al., 2003). In different cells, such as neuronal and hepatoma cells, *in vitro* electrophoretic mobility shift assays, the differential binding activity of two alleles and multiple transcription factors has been evident (Artiga et al., 1998; Lambert et al., 2004; Maloney et al., 2010). After death, biochemical analysis of brain tissue demonstrated its regulatory role in the neural system; T/T genotype was associated with decreased *APOE* expression (Lambert et al., 1998a, 2005). In a case-control study, compared with the rs405509 T/G+G/G group, the rs405509 T/T homozygote increased the risk for developing AD (Lambert et al., 2002).

Resting-state functional magnetic resonance imaging (rs-fMRI) has become a reliable tool for studying brain function (Biswal et al., 1995; Fox and Raichle, 2007). Zhang et al. (2020) found that T/T carriers showed an accelerated age-related increase in functional activation in the left postcentral gyrus compared with G-allele carriers, which demonstrate that the rs405509 T/T allele of APOE causes an age-related brain functional decline in nondemented elderly people. Ma et al. (2016a) found that the APOE-rs405509 interaction impairs elderly’s cognitive performance through brain functional network. Additionally, there are also studies that report rs405509 polymorphism affects brain’s structure (Shu et al., 2015; Ma et al., 2016b; Chang et al., 2017). Although most analytical techniques such as graph theory, independent component analysis (ICA), seed-based functional connectivity (FC) have described the function of the brain network, these methods cannot completely address local neural changes. Currently, several approaches have been recommended to describe the local characteristics using rs-fMRI data, that is, regional homogeneity (ReHo; Zang et al., 2004), amplitude of low frequency fluctuation (ALFF; Zang et al., 2007), fractional amplitude of low frequency fluctuation (Zou et al., 2008), percent amplitude of fluctuation (PerAF; Jia et al., 2020) and degree centrality (DC; Buckner et al., 2009).

ReHo is proposed to measure the functional synchronization between a given voxel and its neighbor voxels (Zang et al., 2004). ALFF, defined as the mean amplitude of fluctuations within the range of low frequency, directly characterizes each voxel’s spontaneous activity (Zang et al., 2007; Zuo et al., 2010). Fractional amplitude of low frequency fluctuations (fALFF), a ratio of the ALFF within a specific low frequency band to the total BOLD fluctuation amplitude within the full frequency band, can be regarded as a standardized ALFF-like metric at the single voxel level and is theoretically a scale-independent method (Zou et al., 2008). PerAF is the percentage of BOLD fluctuations relative to the mean BOLD signal intensity for each time point and averaging across the whole time series, which has better test-retest reliability, both intra- and inter-scanners (Zhao et al., 2018; Jia et al., 2020). DC is proposed to evaluate intrinsic functional connectivity across the whole brain to reflect the functional network’s architecture in a voxel-wise manner (Buckner et al., 2009). These metrics delineate brain functional properties from distinct perspectives. They have been frequently used to study brain functional abnormalities in various neuropsychiatric disorders (Zang et al., 2007; Wu et al., 2009; Hoptman et al., 2010; Paakki et al., 2010; Liang et al., 2011; Premi et al., 2014; Dai et al., 2015; Ma et al., 2019).

In the present study, we employed rs-fMRI to investigate the rs405509’s effects on local brain function in non-demented aging. Considering rs405509 as a promoter that can regulate the expression of APOE, the T to G substitution at rs405509 led to an increase of 169% in promoter activity. We divided the subjects into two groups by rs405509 genotype (TT vs. GG/GT) for further analyzing the differences in neuroimaging. To better understand the polymorphism’s impact on the neural system, it is crucial to enroll non-demented aging individuals, which may help clarify how this polymorphism regulates the risk of developing AD. Specifically, we aimed to determine whether and how rs405509 influences the local function using distinct imaging metrics (ReHo, ALFF, fALFF, PerAF, and DC) and whether these local alterations would be related to the clinical features of the participants. Building on prior literature, we hypothesized that rs405509 T/T allele might cause selective degeneration in specific brain regions functionally in the preclinical stage, and such degeneration may lead to changes in the brain’s cognitive function.

## Materials and Methods

### Participants

A total of 79 non-demented participants were included in the present study. All subjects came from the Beijing Aging Brain Rejuvenation Initiative (BABRI) study, Han Chinese and right-handed. Participants who meet the following criteria were eligible to participate in our research: (1) a score of 24 or higher on the Mini-Mental Status Examination (MMSE); (2) age of 50–80 years; and (3) Clinical Dementia Rating of 0. The exclusion criteria were as follows: (1) abnormalities in the brain structure (non-cerebrovascular injury) revealed by MRI examinations, such as tumors, subdural hematoma, and contusion caused by traumatic brain injury; (2) history of addictions, psychiatric or neurologic disease, or treatments that would affect cognitive function; (3) extensive vessel diseases, such as subcortical or cortical infarcts and watershed infarcts; (4) diseases with white matter lesions, such as multiple sclerosis and normal pressure hydrocephalus; and (5) any contraindications for MRI. The Ethics Committee of The Affiliated Hospital of Qingdao University reviewed and approved the research involving human participants. All subjects gave written informed consent.

### Genotyping

For each participant, we uniformly genotyped the rs405509 polymorphism using a Custom TaqMan SNP Genotyping Assay (Applied Biosystems, Foster City, CA, United States). Another two SNPs, rs7412 and rs429358, which jointly form the *APOE ε*2 (with the rs429358-rs7412 haplotype of T-T), ε3 (T-C), and ε4 alleles (C-C), were also genotyped. The sample success rates for all three SNPs were 100%, and the reproducibility of all the genotyping was 100% according to a duplication analysis of at least 10% of the genotypes. Based on our sample, the rs405509 polymorphism did not show significant deviations from Hardy-Weinberg equilibrium (*p* > 0.5). We divided all subjects into two groups based on their rs405509 genotype: 44 G-allele (including 20 G/T and 24 G/G genotype carriers) and 35 T/T carriers.

### Neuropsychological Testing

To assess cognitive functions, all participants received a battery of neuropsychological tests, including Minimum Mental State Examination (MMSE; Folstein et al., 1975), and other representative neuropsychological tests evaluating definite cognitive functions in the study of aging, including: (1) Clock Drawing Test (CDT; Ishiai et al., 1993), (2) Auditory Verbal Learning Test (AVLT) Delayed Recall (Rosenberg et al., 1984), (3) Boston Naming Test (BNT; Knesevich et al., 1986), (4) Trail Making Test A (TMT-A; Gordon, 1972), (5) Trail Making Test B (TMT-B; Gordon, 1972), (6) Symbol Digit Modifying Test (SDMT; Sheridan et al., 2006), and (7) Stroop Color-Word Test (Stroop; Koss et al., 1984).

### MRI Data Acquisition

The resting-state fMRI data were acquired from a GE Signa HDX 3.0 Tesla scanner at the Affiliated Hospital of Qingdao University, China. To reduce head motion and the impact of scanner noise, we used noise-reducing headphones and foam padding. The subjects were instructed to keep awake with their eyes closed and stayed still as much as possible.

The rs-fMRI data were acquired using an echo-planar imaging sequence: 33 axial slices, repetition time (TR) = 2,000 ms, echo time (TE) = 30 ms, slice thickness = 3.5 mm, flip angle = 90°, matrix = 64 × 64, 240 volumes and field of view (FOV) = 200 mm × 200 mm.

High-resolution T1-weighted images were acquired by using magnetization-prepared rapid gradient-echo (MPRAGE) sequence: 176 sagittal slices, TR = 1,900 ms, TE = 3.44 ms, voxel size: 1 mm × 1 mm × 1 mm, acquisition matrix = 256 × 256, slice thickness = 1 mm, FOV = 256 mm × 256 mm.

### fMRI Data Preprocessing

Resting-state BOLD fMRI data were preprocessed using Data Processing & Analysis for Brain Imaging (DPABI_V4.0) (Yan et al., 2016) based on MATLAB R2014a. (1) The first ten time points were removed to allow the subjects to adapt to the scanning environment and the signal to reach equilibrium; (2) the remaining volumes were corrected for the acquisition time delay between distinct slices; (3) realignment was performed to correct the head motion, all participants’ translational or rotational motion parameters were less than 2 mm or 2° throughout the scanning; frame-wise displacement (FD), which indexes the volume-to-volume changes in head position, is also calculated; (4) in the spatial normalization step, the high-resolution structural images were firstly co-registered to the mean functional images; then we used Diffeomorphic Anatomical Registration Through Exponentiated Lie algebra (DARTEL) algorithm to segment and normalize the co-registered structural images to the Montreal Neurological Institute (MNI) space; finally, the functional images were transformed to the MNI space using the same transformation matrices; (5) smoothing normalized data with a 6 mm full-width at half maximum(FWHM) Gaussian kernel; (6) linear detrending; (7) several nuisance covariates (head motion effect based on the Friston-24 model, the spike volumes with FD > 0.5, the white matter signal, and the cerebrospinal fluid signal) were regressed out from the data (Friston et al., 1996; Power et al., 2012); (8) the data were then band-pass filtered (0.01–0.08 Hz). No pre-processing filtering used for ALFF and fALFF. The fMRI data for ReHo and DC calculation were not smoothed during the pre-processing procedure.

### ALFF Analysis

After preprocessing, the time courses were converted to the frequency domain using a fast Fourier transform, and the power spectrum was obtained by square-rooted fast Fourier transform and averaged across 0.01–0.08 Hz at each voxel. The averaged square root was considered as the ALFF. To decrease global effects of variability across subjects, the ALFF of each voxel was further divided by the global mean ALFF.

### fALFF Analysis

After obtaining the power spectrum, the square root was calculated at each frequency of the power spectrum and the mean square root was acquired across 0.01–0.08 Hz band for each voxel. At last, fALFF in each voxel was divided by mean fALFF of the global brain within a full brain mask to standardize for voxels in the whole brain.

### PerAF Analysis

The PerAF of each voxel was calculated as follows,


(1) $$P\,e\,r\,A\,F=\frac{1}{n}\,\sum_{i=1}^{n}\left|\frac{X_{i}-\mu }{\mu }\right|\times 100\%$$

$$\mu =\frac{1}{n}\,\sum_{i=1}^{n}X_{i}$$

where *X*_*i*_ is the signal intensity of the *i*th time point, *n* is the total number of time points of the time series, and μ is the mean value of the time series.

### ReHo Analysis

The functional data without spatial smoothing were used to calculate ReHo. Kendall’s coefficient of concordance (KCC) was used to calculate the synchronization of the time series between a given voxel and its nearest neighbors.


$$W=\frac{\sum {\left(R_{i}\right)}^{2}-n\,{\left(\bar{R}\right)}^{2}}{\frac{1}{12}\,K^{2}\,\left(n^{3}-n\right)}$$

where *W* is the KCC among given voxels, which ranges from 0 to 1; *R*_*i *_is the sum rank of the time point; $\bar{R}$ is the average value of *R*_*i*_; *n* represents the length of time series, and *K* is the size of the current cluster (*K* = 27). To achieve standardization, the ReHo value of each voxel was then divided by the global mean ReHo value. Ultimately, the ReHo maps spatially smoothed with a 6 mm FWHM Gaussian kernel to reduce noise.

### DC Analysis

Degree centrality computation procedure was conducted using fMRI data without smoothing. Pearson’s correlation coefficients were calculated between each voxel and each other voxel in the entire brain, and a gray matter functional connectivity matrix for each subject was acquired (Zuo et al., 2012). For a given voxel, weighted DC was calculated as the sum of FC over a threshold of 0.6 between that voxel and all other voxels within the entire gray matter. Next, the weighted DC of each voxel was divided by the global mean weighted DC of each subject to achieve standardization. Finally, we spatially smoothed the weighted DC maps with a 6 mm FWHM Gaussian kernel. We also analyzed weighted DC differences between T/T carriers and G allele carriers across different r-value thresholds. The specific details are in the Supplementary Materials.

### Statistical Analysis

The differences between T/T carriers and G-allele carriers in age and education were tested with two-sample *t*-tests. The gender difference was examined with the Pearson Chi-Square test.

Two sample *t*-tests were applied to compare the ALFF, fALFF, PerAF, ReHo, and DC maps between T/T carriers and G-allele carriers. To reduce potential effects on the results, we included individual sex, age, education and mean FD as nuisance covariates. Multiple comparisons were corrected using a Gaussian Random Field (GRF) correction with a cluster-defining threshold of *p* < 0.001 and a corrected significance of *p* < 0.05 in the cluster level. The above-described statistical analyses were done using DPABI (Yan et al., 2016). If any measure exhibited a between-group difference in a cluster (defined as a region of interest [ROI]), a Pearson correlation analysis was performed in all subjects to evaluate its associations with the neuropsychological test scores in an ROI-wise manner. At the threshold of *p* < 0.05, the correlation was considered significant.

## Results

### Cognitive Characteristic

The demographic and neuropsychological data are presented in Table 1. There were no significant differences in age, gender, or educational years between rs405509 T/T genotype and G-allele carriers groups.

**TABLE 1:** Demographic and neuropsychological data of the sample.

**Characteristics**	**T/T carrier**	**G-allele carrier**	**Statistics**	***p* Value**
Number of subjects	35	44		
Age(years)	65.31 ± 2.39	64.80 ± 2.38	t = 0.962	0.339^*a*^
Education(years)	8.83 ± 2.69	9.48 ± 1.53	t = -1.270	0.210^*a*^
Gender(M/F)	15/20	19/25	χ^2^ = 0.001	0.977^*b*^
**General cognition**				
MMSE	25.20 ± 1.02	28.57 ± 1.11	t = 13.879	<0.001^*a*^
**Memory**				
AVLT-Delayed Recall	3.74 ± 1.07	4.34 ± 1.03	t = -2.520	0.014^*a*^
AVLT-T	22.00 ± 2.98	23.75 ± 4.13	t = -2.184	0.032^*a*^
ROCF-delay recall	13.74 ± 3.45	14.41 ± 2.96	t = 0.923	0.359^*a*^
**Attention**				
SDMT	28.20 ± 8.87	33.59 ± 11.47	t = -2.288	0.025^*a*^
TMT-A time(s)	76.43 ± 29.56	67.75 ± 14.87	t = 1.584	0.120^*a*^
**Executive Function**				
SCWT-C time	78.94 ± 27.68	74.84 ± 20.13	t = 0.736	0.465^*a*^
SCWT-C right	43.89 ± 5.11	45.02 ± 4.15	t = -1.066	0.290^*a*^
TMT-B time(s)	203.20 ± 72.60	186.41 ± 57.19	t = 1.150	0.254^*a*^
**Language**				
BNT	21.66 ± 2.66	22.18 ± 2.71	t = -0.863	0.391^*a*^

*The data are shown as the mean ± standard deviation. M, male; F, female; MMSE, Mini-Mental State Examination; AVLT, Auditory Verbal Learning Test; AVLT-T, AVLT-total; ROCF, Rey-Osterrieth Complex Figure; SDMT, Symbol Digit Modalities Test; TMT, Trail Making Test; SCWT, Stroop Color and Word Test; BNT, Boston Naming Test.*

*^*a*^The *p* values were obtained by two-sample *t*-tests.*

*^*b*^The *p* value was obtained by chi-square test.*

Compared with the G-allele carriers, the T/T allele carriers showed decreased MMSE (*p* < 0.001), AVLT-Delayed Recall (*p* = 0.014), AVLT-T (*p* = 0.032), and SDMT (*p* = 0.025), indicating a decline in the general cognitive status, memory, and attention.

### Intergroup Differences in ALFF, fALFF, PerAF, ReHo, and DC

**FIGURE 1:**
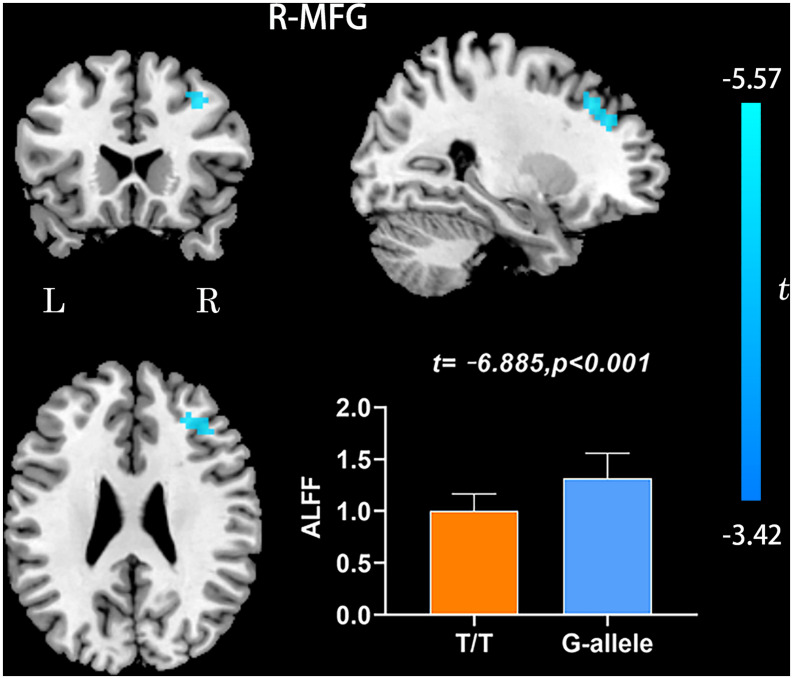
ALFF differences between T/T carriers and G-allele carriers. Error bar represents the standard deviation. ALFF, amplitude of low frequency fluctuation; L, left; R, right.

**FIGURE 2:**
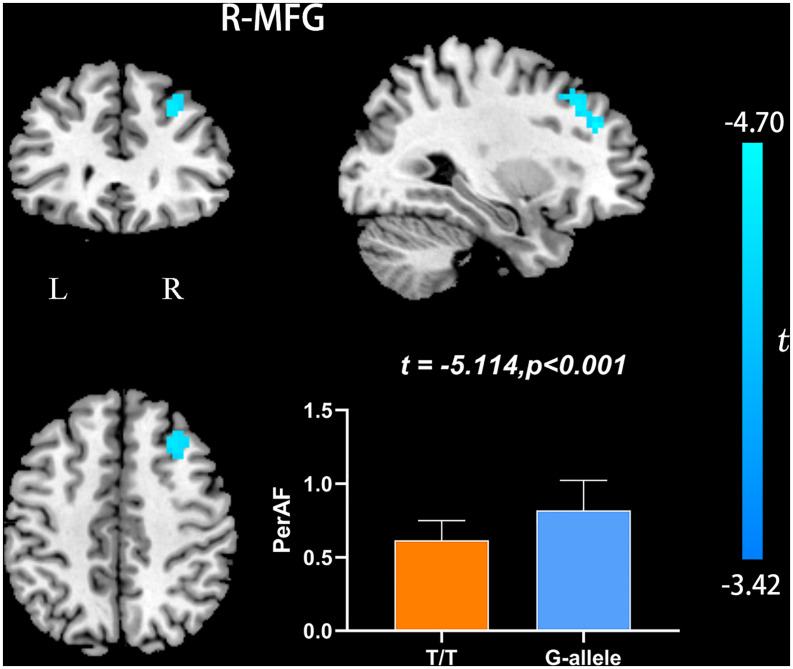
PerAF differences between T/T carriers and G-allele carriers. Error bar represents the standard deviation. PerAF, percent amplitude of fluctuation; L, left; R, right.

**FIGURE 3:**
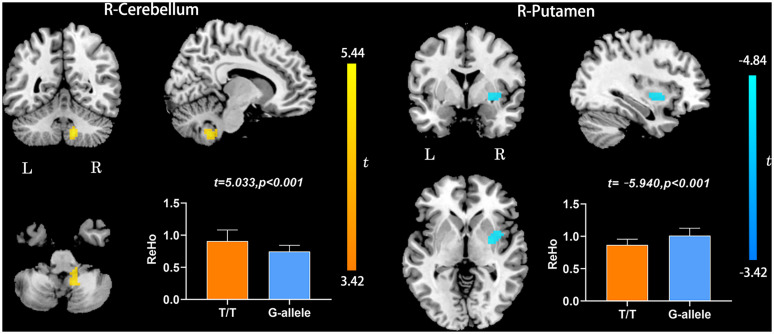
ReHo differences between T/T carriers and G-allele carriers. Error bar indicates the standard deviation. Abbreviations: ReHo, regional homogeneity; L, left; R, right.

**FIGURE 4:**
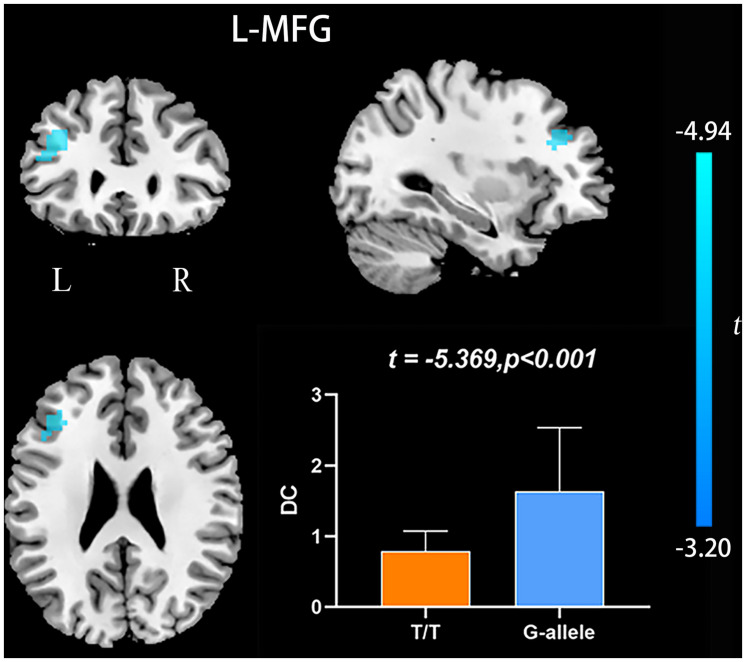
DC differences between T/T carriers and G-allele carriers. Error bar represents the standard deviation. DC, degree centrality; L, left; R, right.

Compared with G-allele carriers, T/T carriers exhibited decreased ALFF in the right middle frontal gyrus (Table 2 and Figure 1), decreased PerAF in the right middle frontal gyrus (Table 2 and Figure 2), increased ReHo in the right cerebellar tonsil and decreased ReHo in the right putamen (Table 2 and Figure 3), and decreased DC in the left middle frontal gyrus (Table 2 and Figure 4) (*p* < 0.05, GRF corrected). For fALFF, no cluster survived after multiple comparisons (*p* > 0.05, GRF corrected).

**TABLE 2:** Brain regions showing ALFF, PerAF, ReHo, and DC differences between the two groups.

			**MNI coordinate (mm)**		
**Regions**	**Cluster size (voxels)**	**Peak T value**	**X**	**Y**	**Z**
**ALFF**					
Middle Frontal Gyrus (R)	114	−5.5769	30	36	30
**PerAF**					
Middle Frontal Gyrus (R)	58	−4.7067	30	36	30
**ReHo**					
Cerebellar Tonsil (R)	79	5.4451	12	−48	−45
Putamen (R)	69	−4.8474	30	−3	−3
**DC**					
Middle Frontal Gyrus (L)	83	−4.9383	−36	33	30

*ReHo, regional homogeneity; ALFF, amplitude of low frequency fluctuation; PerAF, percent amplitude of fluctuation; DC, degree centrality; L, left; R, right.*

### Correlations Between Neuroimaging Parameters and Cognitive Scores

**FIGURE 5:**
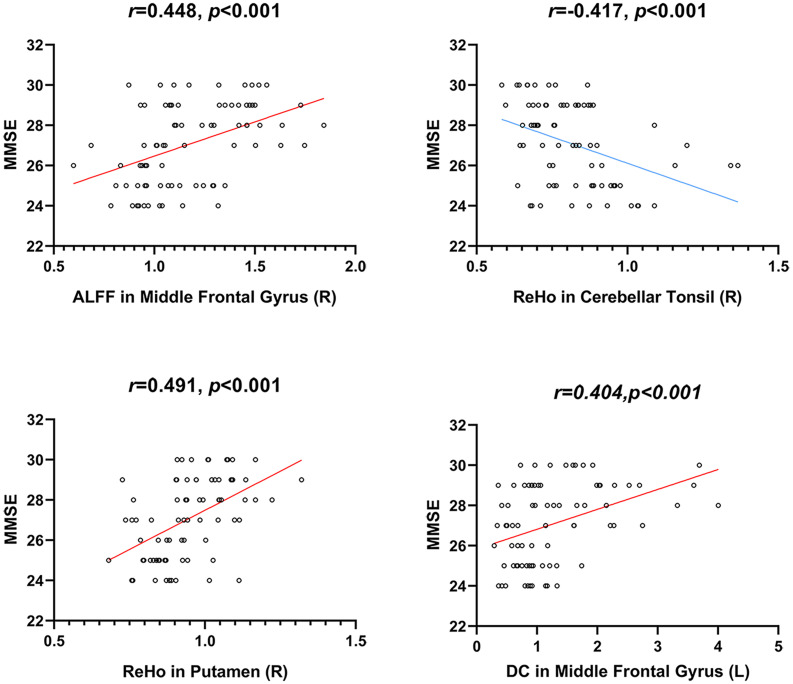
Correlations between local neuroimaging metrics and cognitive performance. MMSE, Mini-Mental State Examination.

We conducted correlation analyses between ROI-based mean imaging values and cognitive scores. Correlations were found between MMSE and imaging parameters in significant ROIs of ALFF, ReHo, and DC (Table 3 and Figure 5). No significant correlation was found for other cognitive measures and other local metrics.

**TABLE 3:** Relationship between local neuroimaging metrics and cognitive performance in a ROI-based correlation analysis.

	**ALFF**	**ReHo**		**DC**
**Cognitive tests**	**Right Middle Frontal Gyrus**	**Right Cerebellar Tonsil**	**Right Putamen**	**Left Middle Frontal Gyrus**
MMSE	*r* = 0.448 *p* < 0.001*	*r* = -0.417 *p* < 0.001*	*r* = 0.491 *p* < 0.001*	*r* = 0.404 *p* < 0.001*

ReHo, regional homogeneity; ALFF, amplitude of low frequency fluctuation; DC, degree centrality; ROI, region of interest; MMSE, Mini-Mental State Examination. *p < 0.05.

## Discussion

The ALFF, fALFF, PerAF ReHo, and DC represent the local brain function from distinct aspects. These local metrics can identify regional brain abnormalities with great sensitivity. In the current study, we used these local metrics to find local brain functional differences between the T/T carriers and G allele carriers and further analyzed the relationship of these functional alterations with neuropsychological scores. The results showed that compared with the G allele carriers, the T/T carriers manifested decreased ALFF in the right middle frontal gyrus, decreased PerAF in the right middle frontal gyrus, increased ReHo in the right cerebellum posterior lobe, decreased ReHo in the right putamen, and decreased DC in the left middle frontal gyrus. These findings may improve our understanding of the influence of rs405509 on the functional changes of *APOEε4* non-carriers.

Amplitude of low frequency fluctuation reflects the degree of spontaneous neural activity (Zang et al., 2007). In the current study, the right middle frontal gyrus of T/T carriers showed decreased ALFF, which represented reduced spontaneous neural activity within this area. The middle frontal gyrus is thought to be involved in episodic memory retrieval (Buckner et al., 2000; West, 2000; Corbetta et al., 2008), and the performance of neuropsychological tests in T/T carriers also confirmed the fMRI results. Meanwhile, the right middle frontal gyrus may act as a critical node of the ventral and dorsal networks (Fox et al., 2006). Therefore, we speculate that the reduced local neural activity in the right middle frontal gyrus could account for the decrease of memory and attention. In the current study, we also found decreased PerAF in the right middle frontal gyrus, consistent with the ALFF result. PerAF has better test-retest reliability than conventional ALFF and much better than fALFF, both intra- and inter-scanners (Zhao et al., 2018; Jia et al., 2020). However, for the fALFF analysis, no cluster survived after multiple comparisons (*p* > 0.05, GRF corrected). Compared with ALFF, previous researches have shown that fALFF had lower test-retest reliability in gray matter voxels (Zuo et al., 2010; Küblböck et al., 2014). Consequently, fALFF may not be a suitable standardized metric for frequency-specific studies. For a single voxel, PerAF is a more promising metric for resting-state BOLD fMRI signal. Therefore, we believe that the results of ALFF and PerAF can complement each other to increase the credibility and persuasiveness of the results.

Regional homogeneity is a local index that reflects the local synchronization of the BOLD signal. The right cerebellar tonsil showed increased ReHo, while the right putamen showed decreased ReHo. The cerebellar tonsil is a part of the cerebellar vermis, which is crucial in regulating cognition and emotion (Stoodley and Schmahmann, 2009). Here, we found increased ReHo in the right cerebellum tonsil, which could be explained as a compensatory process. The compensatory hypothesis is considered to occur in the process of AD and in people at high risk of AD (Bookheimer et al., 2000). In addition, we found decreased ReHo in the right putamen in T/T carriers relative to the G allele carriers. The putamen is a part of the striatum, projecting to the substantia nigra pars reticulata and internal pallidal segment. Cortico-basal ganglia–thalamocortical circuits are engaged in the control of movement, behavior, and cognition, as well as rewards and emotions (Obeso et al., 2014). This result may further confirm that T/T allele may contribute to a variety of cognitive-behavioral declines.

Degree centrality is the embodiment of voxels’ status and role in the whole brain network, and this measure represents the most representative and reliable indicator of local functional connections (Buckner et al., 2009). The left middle frontal gyrus showed decreased DC, which suggests a reduced significance of this region in the entire brain. Wen and colleagues found that the left middle frontal gyrus participated in word production and indicated that it might serve as a temporal perceptual information storage space (Wen et al., 2017). Andersson and colleagues argued that the left middle frontal gyrus is classified as the executive attention network. The left middle frontal gyrus’ function is related to subtle attention disorders in the elderly (Andersson et al., 2009). Additionally, the middle frontal gyrus also engaged in an individual’s literacy and numeracy (Koyama et al., 2017). Accordingly, we speculate that the reduced DC in the left middle frontal gyrus may indicate the decreased functional connectivity and may lead to attention, language, and cognition impairments in T/T allele carriers.

These local indexes define the functional characteristics of the brain from distinct perspectives and show a step-by-step relationship. ALFF characterizes the spontaneous neural activity intensity of voxels, fALFF can get better default mode network patterns, PerAF has better test-retest reliability, both intra- and inter-scanners (Zhao et al., 2018), ReHo reveals the significance of voxels among nearest other voxels, and DC describes the importance of voxels in the entire brain. Applying these local metrics can identify regional abnormalities with greater sensitivity. For instance, An et al. (2013) found the group differences of ADHD patients and healthy controls using ReHo and ALFF; they found that group differences in ALFF and ReHo metrics were not the same. This finding intimate that these local metrics reveal changes in the local functions of the brain from different angles and complement each other.

Correlation analyses were conducted between neuro psychological scales and ROI-based mean imaging values. We found correlations between MMSE and imaging parameters in ALFF, ReHo, and DC. However, no significant correlation was found for other cognitive tests and other local metircs. MMSE is the most widely used simple cognitive function assessment scale in clinical practice, which has the advantages of simplicity, time-saving, and easy operation (Folstein et al., 1975). It is mainly used for the preliminary screening of various types of cognitive impairment and dementia. It can comprehensively, accurately, and quickly reflect the mental state and cognitive function of subjects. In this study, MMSE has a significant correlation with neuroimaging indicators, which proves that the neuropsychological scale and resting-state fMRI can simultaneously detect the cognitive impairment of subjects. Combining early cognitive tests and non-invasive resting-state functional magnetic resonance can provide early intervention and prevention for high-risk groups. The current study has several limitations. Firstly, the sample size is relatively modest. In the future, more subjects will be enrolled to increase statistical power. Additionally, no MRI data were collected during the follow-up period; thus, we cannot examine the T/T allele’s ongoing effects on brain function in the elderly. Future longitudinal studies are needed to test whether the present method could be applied to supervise T/T allele carriers’ brain alterations. Finally, due to the limitation of the scanning range, some subjects may not cover the whole brain. Among these subjects, the most inferior portion of the cerebellum is the common area that did not cover. We reported the result of ReHo in the cerebellum. Still, its position is not at the most inferior portion of the cerebellum, so it will not be affected by the scanning range.

## Conclusion

In conclusion, this study demonstrated the effect of rs405509 on local brain function. T/T allele may serve as an independent risk factor that can influence brain function in different regions in non-demented aging. These preliminary findings reveal the significant role of *APOE* promoter polymorphism in the brain and provide novel insight into the risk of rs405509 polymorphism altering brain function and regulating AD development in non-demented aging.

## Data Availability Statement

The raw data supporting the conclusions of this article will be made available by the authors, without undue reservation.

## Ethics Statement

The studies involving human participants were reviewed and approved by the Ethics Committee of The Affiliated Hospital of Qingdao University. The patients/participants provided their written informed consent to participate in this study.

## Author Contributions

JS, XL, and RZ designed the study. QW, XM, BH, and HG were responsible for performing the experiments and collecting the data. DW and HZ analyzed the cognitive and magnetic resonance data and wrote the manuscript. All authors contributed to the article and approved the submitted version.

## Conflict of Interest

The authors declare that the research was conducted in the absence of any commercial or financial relationships that could be construed as a potential conflict of interest.
