# Corrigendum: The Effects of rs405509 on *APOE*ε*4* Non-carriers in Non-demented Aging

**DOI:** 10.3389/fnins.2022.928289

**Published:** 2022-05-10

**Authors:** Dongpeng Wu, Han Zhao, Huali Gu, Bin Han, Qingqing Wang, Xu Man, Renliang Zhao, Xuejun Liu, Jinping Sun

**Affiliations:** ^1^Department of Neurology, The Affiliated Hospital of Qingdao University, Qingdao, China; ^2^Department of Radiology, The Affiliated Hospital of Qingdao University, Qingdao, China; ^3^Department of Emergency Internal Medicine, The Affiliated Hospital of Qingdao University, Qingdao, China; ^4^Institute of Integrative Medicine, The Affiliated Hospital of Qingdao University, Qingdao, China

**Keywords:** *APOEε4*, Alzheimer's disease, rs405509, amplitude of low-frequency fluctuations, degree centrality (DC), regional homogeneity (ReHo), fALFF (fractional amplitude of low frequency fluctuations), PerAF

In the original article there was an error in the keyword: “*ApoE*ε*4*”, it has been corrected with: “*APOE*ε*4*.”

In the original article, there was an error in the Funding statement as published. The grant number was given as “2018YFC1315300” but should be “2018YFC1315200.” The corrected Funding statement is included below.

“This study was supported by a grant from the National Key R&D Program of China (2018YFC1315200).”

The authors apologize for this error and state that this does not change the scientific conclusions of the article in any way. The original article has been updated.

## Publisher's Note

All claims expressed in this article are solely those of the authors and do not necessarily represent those of their affiliated organizations, or those of the publisher, the editors and the reviewers. Any product that may be evaluated in this article, or claim that may be made by its manufacturer, is not guaranteed or endorsed by the publisher.

